# Latest Comprehensive Medical Resource Consumption in Robot-Assisted versus Laparoscopic and Traditional Open Radical Prostatectomy: A Nationwide Population-Based Cohort Study

**DOI:** 10.3390/cancers13071564

**Published:** 2021-03-29

**Authors:** Szu-Yuan Wu, Shyh-Chyi Chang, Chang-I Chen, Chung-Chien Huang

**Affiliations:** 1 Department of Food Nutrition and Health Biotechnology, College of Medical and Health Science, Asia University, Taichung 413, Taiwan; szuyuan@tmu.edu.tw; 2 Big Data Center, Lo-Hsu Medical Foundation, Lotung Poh-Ai Hospital, Yilan 256, Taiwan; 3 Division of Radiation Oncology, Lo-Hsu Medical Foundation, Lotung Poh-Ai Hospital, Yilan 256, Taiwan; 4 Department of Healthcare Administration, College of Medical and Health Science, Asia University, Taichung 413, Taiwan; 5 Cancer Center, Lo-Hsu Medical Foundation, Lotung Poh-Ai Hospital, Yilan 256, Taiwan; 6 Graduate Institute of Business Administration, Fu Jen Catholic University, Taipei 242062, Taiwan; 7 Centers for Regional Anesthesia and Pain Medicine, Wan Fang Hospital, Taipei Medical University, Taipei 110, Taiwan; 8 Department of Urology, Lo-Hsu Medical Foundation, Lotung Poh-Ai Hospital, Yilan 256, Taiwan; c093006@mail.pohai.org.tw; 9 Faculty of Medicine, National Yang-Ming University School of Medicine, Taipei 11221, Taiwan; 10 Department of Health Care Administration, College of Management, Taipei Medical University, Taipei 110, Taiwan; dcchen@tmu.edu.tw; 11 International Ph.D. Program in Biotech and Healthcare Management, Taipei Medical University, Taipei 110, Taiwan

**Keywords:** medical reimbursement, hospitalization, robotic radical prostatectomy, open radical prostatectomy, laparoscopic radical prostatectomy

## Abstract

**Simple Summary:**

Few studies have evaluated long-term medical monetary cost in patients with prostate cancer (PC) receiving open radical prostatectomy (ORP), laparoscopic radical prostatectomy (LRP), or robot-assisted radical prostatectomy (RARP). To the best of our knowledge, this is the largest and longest follow-up study to examine medical monetary cost in patients with PC undergoing ORP, LRP, or RARP. After adjustment for confounders, the medical monetary cost in the RARP group was the least compared with that in the ORP and LRP groups.

**Abstract:**

Background: Few studies have evaluated long-term medical monetary cost in patients with prostate cancer (PC) receiving open radical prostatectomy (ORP), laparoscopic radical prostatectomy (LRP), or robot-assisted radical prostatectomy (RARP). To the best of our knowledge, this is the largest and longest follow-up study to examine medical monetary cost in patients with PC undergoing ORP, LRP, or RARP. After adjustment for confounders, the medical monetary cost in the RARP group was the least compared with that in the ORP and LRP groups. Purpose: To estimate long-term medical resource consumption among patients with prostate cancer (PC) receiving open radical prostatectomy (ORP), laparoscopic radical prostatectomy (LRP), or robot-assisted radical prostatectomy (RARP). Patients and Methods: Participants were men enrolled in the Taiwan Cancer Registry with localized PC diagnosis who received radical prostatectomy. After adjustment for confounders, a generalized linear mixed model was used to determine significant differences in the number of urology outpatient clinic visits required, proportion of patients being hospitalized for urinary diseases or surgical complications, and medical reimbursement for urinary diseases or surgical complications following ORP, LRP, or RARP in the first, second, and third years. *Results:* No differences were observed in the median number of urology outpatient clinic visits between the three types of surgical modalities up to the second year after ORP, LRP, and RARP (median: 15, 10, and seven visits, respectively; *p* < 0.001), but significant differences were observed in the third year. Similarly, with RARP (10.9% versus 18.7% in ORP and 9.8% in LRP; *p* = 0.0014), the rate of hospitalization for urinary diseases or surgical complications decreased in the third year. Medical reimbursement for urinary diseases or surgical complications reduced after RARP compared with that for ORP and LRP, with approximately 22% reduction in the first year (*p* = 0.0052) and 20–40% reduction in the third year (*p* value = 0.0024). Conclusions: Medical resource consumption in the RARP group was less compared with those in the ORP and LRP groups.

## 1. Introduction

Prostate cancer (PC) has the highest incidence among male cancers in the United States and is the sixth cause of death among men in Taiwan [1,2,3,4]. In the United States, one in nine men is diagnosed with PC during his lifetime [5]. PC is most likely to develop in older men (≥65 years) and in African American men, and it rarely occurs in men <40 years old [5]. Although it may affect daily activities, it does not shorten life expectancy or reduce quality of life [6]. Treatment is often not required due to its unique characteristics, but if required, it is either for curing or for controlling the spread of the disease [6].

Radical prostatectomy (RP) has been widely recognized as standard treatment for localized PC [3,4]. Currently, three common approaches are available for this surgery: robot-assisted RP (RARP), laparoscopic RP (LRP), and traditional open RP (ORP) [3,4]. LRP carries a rather long learning curve. For this reason, its adoption was not universal and many urologists stuck to the still ‘gold standard’ of ORP. With the advent of RARP this was different. RARP does have a shorter learning curve, it can be adopted by surgeons without laparoscopic experience, and its control of the instruments is less tiring and easier than laparoscopy [7]. This allowed many experienced open surgeons to switch to robotic surgery much more easily than the transition to laparoscopic surgery had been. Probably for this reason, robotic surgery made its advance into urological surgery much more quickly and easily than laparoscopy ever did [8]. Since its clinical introduction in Europe in 1999, the da Vinci system has opened up a new era in minimally invasive surgery [9,10]. It allows refined microsurgical preparation and suturing, easier and better than laparoscopy ever did, and provides excellent endoscopic vision also better than in laparoscopy. As a result, RARP became popular compared to LRP and ORP.

The highlighted superiority of RARP over LRP and ORP in terms of blood loss, complication incidence, incontinence, and sexual function loss has been established over the past decades [11,12,13,14,15,16,17,18,19,20]. However, little evidence is available on the mechanism through which RARP affects the empirical postoperative complications and long-term medical resource consumption, such as the number of urological outpatient visits required, subsequent hospitalizations, and medical costs due to surgical complications incurred after RP over LRP and ORP. Although RP is a proven method for PC treatment, treatment method selection among RARP, LRP, and traditional ORP and the suitable approach with the lowest intraoperative and postoperative complications are usually not only based on a clinical decision but also influenced by affordability, accessibility, and a lack of empirical outcome evidence, particularly in terms of medical resource consumption. Therefore, our study aimed to determine the true and real-world medical resource consumption of the different surgical techniques of RP.

Studies comparing RP in terms of acute and chronic medical resource consumption are scant, and relevant findings have been inconsistent [21,22,23,24,25]. Hence, we conducted a complete retrospective cohort study to explore the long-term medical resource consumption of the three surgical modalities in the first, second, and third years based on: (1) the number of urological outpatient clinic visits following the surgery, (2) subsequent related hospitalizations, and (3) total medical costs associated with postsurgical complications. The real-world information will serve as reference for establishing future health policies in the department of health and welfare.

## 2. Patients and Methods

### 2.1. Data Source

Data were extracted from the Nationwide Taiwan Cancer Registry database (TCRD) and Taiwan National Health Insurance (NHI) Research Database (NHIRD). The TCRD was established in 1979 and contains the data of 97% of cancer cases in Taiwan [26]. Furthermore, it is linked with the NHIRD through encrypted patient identifiers. The NHIRD includes all medical claims data on disease diagnoses, procedures, drug prescriptions, demographics, and enrollment profiles of all beneficiaries [27]. The TCRD of the Collaboration Center of Health Information Application contains detailed patient information, such as clinical stages, medical cost, treatment complication-related management, surgical procedures, techniques, radiotherapy, chemotherapy regimens and follow-up visits, and medical cost [3,4,28,29,30,31,32,33]. The medical marketplace is dynamic and constantly advancing [34]. RARP was introduced to Taiwan in 2004, and 5 years after popular positive feedback from the urology community, Taiwan NHI initiated RARP reimbursement in 2014 [35], and thus, every procedure from 2015 onward must be filed for receiving claims; therefore, we can trace RARP through the NHIRD and TCRD [3,4]. Moreover, in 2014, the most common brand of RARP, Da Vinci Xi, was approved by Taiwan Food and Drug Administration and has been applied in Taiwan since then [35]. Because RARP reimbursement began in 2014, this study targeted all patients who underwent RP in 2015 and investigated them for up to 3 years, with follow-up ending on December 31, 2018.

### 2.2. Study Cohort

The cohort consisted of patients who received a diagnosis of resectable PC and underwent RP between 1 January 2015, and 31 December 2015, who were identified from the TCRD. The index date was the surgery date. The follow-up duration was the period from the index date until December 31, 2018. The study protocols were reviewed and approved by the Institutional Review Board of Tzu-Chi Medical Foundation (IRB109-015-B). Patient diagnoses were confirmed through a review of their pathological data, and patients who received a new diagnosis of resectable PC and underwent RP were confirmed to have no other cancer or distant metastasis. The RP type we examined was the surgical procedure to remove the entire prostate gland and surrounding lymph nodes as treatment for men with localized PC [36]. The inclusion criteria were as follows: a diagnosis of resectable PC, an indication for RP, age ≥20 years, and histologically confirmed PC in clinical stages T1–4 without distant metastasis (using the staging system of the American Joint Committee on Cancer). In our study, T1 means that cancer was found during prostate examination. After pathological confirmation of PC through biopsy, patients with PC chose RP, radiotherapy, or active surveillance depending on criteria of the National Comprehensive Cancer Network risk groups and the patient’s expected survival time [37]. Here, pT1 was defined as the combined data of pathological proof or RP and TCRD records. Those with a history of cancer before PC diagnosis, unknown clinical or pathological stage, unknown D’Amico risk classification, unknown Gleason score, unknown postoperative Gleason Grade group, missing data on the preoperative prostate-specific antigen (PSA) concentration, clinical node-positive PC, unclear margin status, and nonadenocarcinoma histology were excluded. To compare their outcomes, patients undergoing open, laparoscopic, and robotic RP were assigned to groups 1, 2, and 3, respectively.

### 2.3. Covariates and Endpoints

The main independent variable in this study was the RP type: ORP, LRP, or RARP. Other covariates were age, clinical and pathological T stage, Gleason score, preoperative PSA in nanogram per millimeter, Damico risk classification, and hospital level (academic or nonacademic). Dependent variables were: (1) the number of urology outpatient clinic visits required, (2) the proportion of patients being hospitalized, and (3) the medical costs for surgical complications.

### 2.4. Statistical Analysis

This population-based retrospective cohort study was conducted using a generalized linear mixed model with multivariate analysis with adjustment for covariates, such as age, clinical and pathological T stage, Gleason score, preoperative PSA, Damico risk classification, hospital level, and surgical modality, for all patients who underwent RP in 2015. We fit a generalized linear mixed model with random intercept for patient clustering within hospitals and Type III tests of fixed effects. Therefore, there is only one *p* value shown. Patient characteristics were first described according to the surgical modality by using descriptive statistics such as mean and standard deviation for normal continuous data, medians and interquartile ranges for nonnormal continuous data, and number and proportions for categorical data. Student’s *t* test, analysis of variance, and their nonparametric counterpart tests were used, as appropriate. Two types of multivariate mixed models accounting for patient clusters in hospitals were fitted to ascertain the effect of RARP on the outcomes: (1) linear models for continuous outcomes, number of urology outpatient clinic visits, and medical costs for the treatment of surgical complications and (2) a logistic regression model for hospitalization for surgical complications, with adjustment for age, clinical and pathological T stage, Gleason score, preoperative PSA, Damico risk classification, and hospital level. Significance levels were set at 5%.

## 3. Results

### 3.1. Clinicopathological Characteristics

Of the 1407 patients included in the study, 315, 276, and 816 had received ORP, LRP, and RARP, respectively. The mean follow-up duration after the index date was 36.67 months, with a standard deviation of 4.63 months. No statistically significant differences were observed in age, clinical T-stage, pathological T-stage, Gleason score, Gleason grade group, preoperative PSA concentration, D’Amico risk classification, hospital level, follow-up duration, and death (Table 1).

### 3.2. Number of Urology Outpatient Clinic Visit Stratified by RP Method

Table 2 shows the numbers of outpatient clinic visits per patient stratified by surgical modality. Although no differences were observed in the median or mean clinic visit numbers across the three types of surgical modalities up to 2 years after ORP, LRP, and RARP (median of 15, 10, and 7 visits, respectively; *p* < 0.001), a significant reduction was observed in the third year.

### 3.3. Hospitalization for Urinary Diseases or Surgical Complications Stratified by RP Method

RARP (10.9% versus 18.7% in ORP and 9.8% in LRP) decreased the hospitalization rate due to urinary diseases or surgical complications (Table 3). No significant differences were observed among ORP, LRP, and RARP in terms of hospitalization in the first 2 years (year 1: 40.3%, 40.9%, and 39.0%, respectively, *p* = 0.8799; year 2: 25.7%, 22.1%, and 22.9%, respectively, *p* = 0.6151) in Table 3. However, major differences were observed from year 3 onward (18.7%, 9.8%, and 10.9%, respectively, *p* = 0.0014).

### 3.4. Medical Reimbursement for Urinary Diseases or Surgical Complications Stratified by RP Method

Treatment costs due to surgical complications were less after RARP than after both ORP and LRP, with approximately 22% reduction in the first year (*p* = 0.0052) and 20–40% reduction in the third year (*p* = 0.0024) (Table 4). This study showed that 3 years’ total medical claim amounts of ORP, LRP, and RARP were New Taiwan Dollars (NTD) 421030, NTD 384432, and NTD 315,399 in terms of the mean (*p* = 0.0029; Table 4) and NTD 264323, NTD 241373, and NTD 202,781 in terms of the median, respectively. RARP could save NTD 105631, which was half of the operation cost at that time (Table 4). Bar graph of generalized linear mixed model of medical reimbursement for urinary diseases or surgical complications stratified by the three techniques as Supplemental Figure S1.

## 4. Discussion

Some studies have shown that the total actual costs were significantly higher with RARP than with LRP or ORP; this was attributed to the robotic equipment and supplies instead of medical resource consumption, such as the number of urology outpatient clinic visits, hospitalization for urinary diseases or surgical complications, or medical reimbursement for urinary diseases or surgical complications [21,22,23,24,25]. Some studies have shown that patients had fewer complications and a decreased frequency of complications as well as shorter hospital-stay and fewer blood transfusions after LRP or RARP than after ORP [11,13,38]. However, data regarding the long-term medical resource consumption of the three surgical techniques of RP perioperatively and postoperatively until now are unavailable. This is the first population-based study of the effect of various surgical modalities on the number of urological clinic visits, hospitalization rate, and medical costs for surgical complications (Table 2, Table 3 and Table 4). RARP significantly reduced the number of urology outpatient clinic visits required after RP compared with that after ORP or LRP and effectively decreased the hospitalization rate for urinary diseases or surgical complications as well as subsequent medical reimbursement for urinary diseases or surgical complications compared with both ORP and LRP.

Analyses of large databases indicate that RARP has increased rapidly in popularity, now constituting the modality used in the majority of cases [13,39]. Similarly, RARP is becoming popular in Taiwan because it minimizes side effects [40,41,42]; nonetheless in the previous studies in Taiwan [3,4], there is limited evidence of results in medical cost with this approach. Recently, RARP become the mainstream treatment for localized prostate cancer in Taiwan [43]. Therefore, there were more men with PC in RARP group than LRP or ORP groups (Table 1).

No significant difference was observed during the first two years for all three modalities in terms of the number of urology outpatient clinic visits (Table 2). These findings might be attributable to the routine urology outpatient clinic follow-up protocol that mandates patients to routinely return to the hospital for follow-up after RP for all three techniques, with a higher number of outpatient visits in the first two years compared with that in the third year. The lower urology outpatient clinic visits in the third year might be due to few surgical complications or the subsiding of surgical complications gradually after RP. The all-inclusive Taiwanese health insurance program and extremely low deductible and copayment made the beneficiaries comply totally to the protocol of visiting their doctors on a regular basis, without any access, financial, or disease barriers [44]. In the third year, both RARP and LRP showed significantly lower resource consumption than ORP (median visits were seven, 10, and 15, respectively; *p* < 0.0001; Table 2). This means that the long-term outcome of RARP is superior after stabilized prognosis relative to LRP and ORP. No study has evaluated medical resource consumption in terms of the number of outpatient medical visits for the different techniques of RP, and ours is the first study to show varying medical consumption due to the number of urology outpatient clinic visits among the three surgical techniques of RP (Table 2). The increased number of urology outpatient clinic visits means that chronic surgery-related complications increased after RP. In our study, the numbers of urology outpatient clinic visit decreased after RARP (Table 2); these cost-related findings might be references for establishing government health policies for selecting RARP instead of ORP or LRP. In our previous study, no differences were observed in positive surgical margin rates or biochemical failure-free survival among patients receiving ORP, LRP, or RARP.^3^ Therefore, long-term medical resource consumption should be considered for establishing health policies in the future.

Hospitalizations due to surgical complications were fewer in the third year after RARP or LRP than in the third year after ORP (Table 3), probably because of substantial differences in postoperative complications related to chronic iatrogenic problems in these surgical procedures. Both LRP and RARP appear to be superior in the long-term, with fewer hospitalizations, but not in the short-term. A study in Vattikuti Urology Institute, Henry Ford Health System, reported that RARP has supplanted ORP as the most common surgical approach for RP [13]. Moreover, a previous study demonstrated superior adjusted perioperative outcomes after RARP for virtually all examined outcomes [13]. In addition, another study in the Division of Urologic Surgery, Brigham and Women’s Hospital, showed that in comparison with patients who underwent ORP, those who underwent LRP or RARP had shorter length of stay, fewer respiratory and miscellaneous surgical complications and strictures, and similar postoperative use of additional cancer therapies but more genitourinary complications, incontinence, and erectile dysfunction [11]. The inconsistent data of RARP, LRP, and ORP in some studies might be because LRP and RARP [11] were not considered separately but together as minimally invasive radical prostatectomy (MIRP) [11]. However, LRP and RARP cause different postoperative complications [13], and therefore, they should not be considered together as MIRP for analyzing the total medical resource consumption. Our study might be compatible with the study by Trinh et al., in which LRP and RARP were considered separately [13]. Our findings (Table 3) suggest that RARP may subsequently reduce the number of rehospitalizations related to surgical complications. However, our study findings indicated that these differences were statistically significant only in the third year after RP. This may be explained by differences in chronic postoperative complications among men who have undergone various surgical techniques for RP. Moreover, this is the first study to show that chronic medical resource consumption was significantly different across the different surgical techniques in the third year after RP. This suggests that RARP may subsequently reduce the number of re-hospitalizations related to surgical complications in the long term. Thus, our study findings indicated that these differences were statistically significant only in the third year after RP. Another plausible explanation is that hospitals with a high hospital volume and readily available facilities for ORP may have been dominant in this population-based study [4]. Therefore, in the first two years after surgery, no significant differences were observed across the techniques; across the techniques, perioperative complications were compatible, and similar acute or subacute surgical complications were observed. The hospitalization rate for urinary diseases or surgical complications was nonsignificantly lower for RARP than for LRP and ORP in the first year (Table 3). The differences in hospitalization rates for urinary diseases or surgical complications varied over time, and the hospitalization rates of RARP, LRP, and ORP in the first, second, and third years were 39.0%, 40.9%, and 40.3% (*p* = 0.8799); 22.9%, 22.1%, and 25.7% (*p* = 0.6151); and 10.9%, 9.8%, and 18.7% (*p* = 0.0014), respectively. Both RARP and LPP exhibit good complication outcomes and less possibility of re-admission for treatment. Another Cochrane review of two randomized trials with all available short-term outcome data demonstrated that urinary complications, sexual quality, and other life-related outcomes appear similar, overall and serious postoperative complication rates appear similar, and the difference in postoperative pain may be minimal [12]. The outcomes of the Cochrane review are compatible with ours in that no significant differences were observed in the first two years in terms of numbers of urology outpatient clinic visit and hospitalization for urinary diseases or surgical complications (Table 2 and Table 3) [12]. Medical resource consumption was significantly different in the third year because of chronic postoperative complications (Table 2 and Table 3).

In fact, there is no data of rehospitalization rate in the first, second, and third year like ours. Most studies showed data of 90-day re-admission rates between ORP, LRP, and RARP [45,46]. The 90 postoperative days (10%) identified were readmitted, specifically 10% after ORP, 9% after RARP and 11% after LRP [45]. However, our data is the rehospitalization rate in the first year around 40%. Our results of time-intervals were different from the previous studies [45,46]. On the other hand, the 99.6% of Taiwan’s 23.57 million people covered under the government-run National Health Insurance (NHI), a universal health care scheme that ensures every resident has access to quality and affordable medical care. The comprehensive coverage includes both inpatient and outpatient care, prescription drugs, traditional Chinese medicine, dental services and home nursing care [47,48]. NHI enrollment is mandatory for all citizens and foreign residents in Taiwan. Needless or duplicated medical exams and drug prescriptions are some of the main contributors to a huge waste of resources [47,48]. Our data might faithfully show the waste of medical resources among the three RP techniques (a 40% rate of hospitalization in the first year of the patients seem to be high for all three techniques, Table 3). However, there is no bias for the waste of medical resources among the three RP techniques. Therefore, the conclusion of the total medical resource consumption in the RARP group was less in terms of the number of urology outpatient clinic visits, hospitalization for urinary diseases or surgical complications, and medical reimbursement for urinary diseases or surgical complications compared with that of the ORP and LRP groups would not be overturned.

The numbers of outpatient clinic visit (Table 2) cannot stand for the medical cost for relief of complications using prescriptions of drugs, medical procedure, or other medical consumables. Therefore, we design the generalized linear mixed model of medical reimbursement for urinary diseases or surgical complications as Table 4 to clarify the medical cost for urinary diseases or surgical complications. Medical cost analysis by using claims data for all urinary diseases or surgical complications reimbursed in the following 3 years after RP (Table 4) showed that RARP is the most cost-effective choice after surgery in years 1, 2, and 3 separately or aggregated. This is the first nation-wide population-based study to prove that RARP has the best outcomes and the least complications and requires the least medical resources (Table 4). However, no significant difference was observed across the three methods in the second year. However, significant differences in medical reimbursement for urinary diseases or surgical complications were observed in the first and third years, where RARP was associated with significantly reduced medical cost compared with LRP and ORP (Table 4). In a previous study, RARP was associated with reduced hospital stay and blood transfusion during perioperative procedures and reduced postoperative complications [13]. The findings by Trinh et al. are compatible with ours and showed that decreased intraoperative complications (medical cost significantly reduced in the first year) [13] and chronic postoperative complications (medical cost significantly reduced in the third year) in the RARP group might be attributable to fewer hospital stays, fewer blood transfusions, and lower medical reimbursement for urinary diseases or surgical complications in RARP than in LRP and ORP [13]. Ours is the first study to report the total medical reimbursement for urinary diseases or surgical complications in the first, second, and third years and the total three years among ORP, LRP, and RARP. This study could serve as a reference and guide for establishing health policies. Although the cost of RARP at the beginning might be more than that of ORP or LRP due to the equipment involved [21,22,23,24,25], the total medical resource consumption in the RARP group is lower in terms of numbers of urology outpatient clinic visits, hospitalization for urinary diseases or surgical complications, and medical reimbursement for urinary diseases or surgical complications compared with ORP and LRP (Table 2, Table 3 and Table 4).

The strength of our study is that covariates were balanced among ORP, LRP, and RARP. We believe that the bias of complicated or advanced surgical cases in ORP, LRP, and RARP was absent (Table 1). Furthermore, our study had the largest sample size and the longest long-term follow-up (three years) for evaluating medical resource consumption in terms of the number of urology outpatient clinic visit, hospitalization for urinary diseases or surgical complications, and medical reimbursement for urinary diseases or surgical complications among ORP, LRP, and RARP. Our study reported fewer numbers of urology outpatient clinic visits and hospitalization for urinary diseases or surgical complications in the RARP group in the third year than in the first and second years. Moreover, medical reimbursement decreased for urinary diseases or surgical complications in patients with PC who underwent RARP compared with those who underwent ORP or LRP in the first and third years. Thus, decreased medical resource consumption might be due to reduction in not only intraoperative complications but also postoperative complications. The total benefits of lower medical resource consumption in patients with PC receiving RARP compared with those receiving ORP or LRP can be used for establishing further health policies. RARP appears to be better that the other two treatment methods in terms of quality, surgical complications (Table 3), number of revisits (Table 2), and home and self-care costs, and it may even consume lower resources in the long term (Table 2, Table 3 and Table 4). The study results can provide policy makers with sufficient information to reconsider the whole reimbursement scheme from a holistic perspective.

Our study has some limitations. First, it was a retrospective observational study where patients with PC were nonrandomly assigned to each RP type based on the preferences of clinicians. Second, although no significant differences in covariate distribution was observed among ORP, LRP, and RARP (Table 1), unobserved confounders could lead to biased results. Finally, cost data can vary substantially between countries. Hence, our study findings may not be generalized. Despite these limitations, our population-based nationwide analyses provide updated, long-term follow-up information for medical resource consumption for RARP compared with the other RP types. The information regarding medical resource consumption among different RP techniques is crucial for establishing future health policies for surgery in patients with PC.

## 5. Conclusions

The total medical resource consumption in the RARP group was less in terms of the number of urology outpatient clinic visits, hospitalization for urinary diseases or surgical complications, and medical reimbursement for urinary diseases or surgical complications compared with that of the ORP and LRP groups.

## Figures and Tables

**Table 1:** Demographic and clinicopathological characteristics stratified by open, laparoscopic, and robotic RP.

Characteristics		Open RP N = 315		Laparoscopic RP N = 276		Robotic RP N = 816		*p* Value
		n	(%)	n	(%)	n	(%)	
Age (years)	Mean (SD)	66.4	(6.8)	66.8	(6.4)	66.1	(6.7)	0.4661
	Median (IQR)	67	(62–71)	67	(62–72)	66	(62–71)	
	20–59	49	(15.6)	41	(14.9)	130	(15.9)	0.9004
	60–69	165	(52.4)	146	(52.9)	444	(54.4)	
	70+	101	(32.1)	89	(32.2)	242	(29.7)	
Clinical T-stage	cT1	84	(26.7)	75	(27.2)	195	(23.9)	0.2839
	cT2	149	(47.3)	133	(48.2)	436	(53.4)	
	cT3-cT4	82	(26.0)	68	(24.6)	185	(22.7)	
Pathological T-stage	pT1	96	(30.5)	83	(30.1)	237	(29.0)	0.1884
	pT2	152	(48.3)	137	(49.6)	432	(52.9)	
	pT3a	37	(11.7)	30	(10.9)	76	(9.3)	
	pT3b	30	(9.5)	26	(9.4)	71	(8.7)	
Gleason score	2–6	34	(10.8)	37	(13.4)	142	(17.4)	0.0951
	3+4	110	(34.9)	89	(32.2)	274	(33.6)	
	4+3	62	(19.7)	53	(19.2)	160	(19.6)	
	8–10	109	(34.6)	97	(35.1)	240	(29.4)	
Preoperative PSA (ng/mL)	Mean (SD)	15.8	(15.9)	17.6	(17.8)	15.8	(16.6)	0.3483
	Median (IQR)	10.3	(6.9–18.0)	10.4	(7.0–20.5)	10.3	(6.7–17.6)	
Preoperative PSA (ng/mL)	0–5	37	(11.7)	32	(11.6)	94	(11.5)	0.6540
	6–10	110	(34.9)	95	(34.4)	285	(34.9)	
	11–20	86	(27.3)	82	(29.7)	233	(28.6)	
	20+	82	(26.0)	67	(24.3)	204	(25.0)	
D’Amico risk classification	Low	13	(4.1)	15	(5.4)	58	(7.1)	0.1117
	Intermediate	93	(29.5)	69	(25.0)	219	(26.8)	
	High	122	(38.7)	120	(43.5)	338	(41.4)	
	Advanced	87	(27.6)	72	(26.1)	201	(24.6)	
Hospital level	Academic center	258	(81.9)	225	(81.5)	673	(82.5)	0.7251
	Nonacademic center	57	(18.1)	51	(18.5)	143	(17.5)	
Follow-up duration (months)	Mean (SD)	36.1	(4.4)	37.2	(5.0)	36.2	(4.7)	
Death		8	(2.5)	4	(1.4)	11	(1.3)	0.3534

RP, radical prostatectomy; T, tumor; PSA, prostate-specific antigen; SD, standard deviation; IQR, interquartile range Table 2. Generalized linear mixed model of numbers of urology outpatient clinic visits stratified by open, laparoscopic, and robotic RP.

**Table 2:** Generalized linear mixed model of numbers of urology outpatient clinic visits stratified by open, laparoscopic, and robotic RP.

Numbers of Outpatient Clinic Visits		Open RP N = 315		Laparoscopic RP N = 276		Robotic RP N = 816		*p* Value *
First year after RP	Mean (SD)	33.0	(10.5)	32.0	(11.6)	36.0	(11.8)	0.72114
	Median (IQR, Q1–Q3)	23	(17–30)	23	(17–30)	22	(16–31)	
Second year after RP	Mean (SD)	22.8	(12.7)	23.3	(13.0)	22.1	(13.1)	0.9478
	Median (IQR, Q1–Q3)	21	(14–29)	20	(14–30)	19	(13–28)	
Third year after RP	Mean (SD)	16.9	(11.7)	13.1	(12.7)	10.3	(10.6)	<0.0001
	Median (IQR, Q1–Q3)	15	(7–22)	10	(6–19)	7	(5–15)	

* Multivariate model with adjustment of covariates: age, clinical and pathological T stages, Gleason score, preoperative prostate-specific antigen, Damico risk classification, and hospital levels. RP, radical prostatectomy; SD, standard deviation; IQR, interquartile range.

**Table 3:** Generalized linear mixed model of hospitalization for urinary diseases or surgical complications stratified by open, laparoscopic, and robotic RP.

Hospitalization (%)	Open RP N = 315		Laparoscopic RP N = 276		Robotic RP N = 816		*p* Value *
First year after RP	127	(40.3)	113	(40.9)	319	(39.0)	0.8799
Second year after RP	81	(25.7)	61	(22.1)	187	(22.9)	0.6151
Third year after RP	59	(18.7)	27	(9.8)	89	(10.9)	0.0014

* Multivariate model with adjustment of covariates: age, clinical and pathological T stages, Gleason score, preoperative prostate-specific antigen, Damico risk classification, and hospital levels. RP, radical prostatectomy.

**Table 4:** Generalized linear mixed model of medical reimbursement for urinary diseases or surgical complications stratified by open, laparoscopic, and robotic RP.

Medical Cost (NTD)		Open RP N = 315		Laparoscopic RP N = 276		Robotic RP N = 816		*p* Value *
First year after RP	Mean (SD)	230492.4	(140659.6)	232261.9	(141475.8)	181923.1	(125129.5)	0.0052
	Median (IQR, Q1–Q3)	178584	(145267–245037)	177489	(147614–237280)	142619	(110697–193688)	
Second year after RP	Mean (SD)	114771.2	(199274.4)	95077.7	(144789.0)	88050.5	(140079.7)	0.1280
	Median (IQR, Q1–Q3)	53493	(22457–109758)	43428	(23227–93992)	45653	(21740–84986)	
Third year after RP	Mean (SD)	75767.3	(138665.8)	57092.6	(119648.4)	45426.1	(97456.8)	0.0024
	Median (IQR, Q1–Q3)	32246	(10844–71234)	20456	(5192–46936)	14509	(4320–39596)	
3-year total	Mean (SD)	421030	(153387.1)	384432	(13326.2)	315399	(116010.3)	0.0029
	Median (IQR, Q1–Q3)	264323	(14223–131167)	241373	(14427–126241)	202781	(56610–109821)	

* Multivariate model with adjustment of covariates: age, clinical and pathological T stages, Gleason score, preoperative prostate-specific antigen, Damico risk classification, and hospital levels. RP, radical prostatectomy; SD, standard deviation; IQR, interquartile range; NTD, New Taiwan Dollars.

## Data Availability

Restrictions apply to the availability of these data. Data was obtained from Taiwan Ministry of Health and Welfare and are available from S.-Y.W. with the permission of Institutional Review Board of Tzu-Chi Medical Foundation (IRB109-015-B).

## Supplementary Materials

The following are available online at https://www.mdpi.com/article/10.3390/cancers13071564/s1, Figure S1: Bar graph of generalized linear mixed model of medical reimbursement for urinary diseases or surgical complications stratified by open, laparoscopic, and robotic RP.

## Abbreviations

RP	Radical prostatectomy
PC	Prostate cancer
T	Tumor
PSA	Prostate-specific antigen
ORP	Open radical prostatectomy
LRP	Laparoscopic radical prostatectomy
RARP	Robot-assisted radical prostatectomy
TCRD	Taiwan Cancer Registry database
NHI	National Health Insurance
NHIRD	National Health Insurance Research Data
NTD	New Taiwan Dollars
MIRP	Minimally invasive radical prostatectomy

## References

[1] Health Promotion Administration (2017). Taiwan Cancer Registry Annual Report.

[2] Rawla P. (2019). Epidemiology of Prostate Cancer. World J. Oncol..

[3] Chang S.C., Chen H.M., Wu S.Y. (2020). There Are No Differences in Positive Surgical Margin Rates or Biochemical Failure-Free Survival among Patients Receiving Open, Laparoscopic, or Robotic Radical Prostatectomy: A Nationwide Cohort Study from the National Cancer Database. Cancers.

[4] Chang S.C., Hsu C.H., Lin Y.C., Wu S.Y. (2021). Effects of 1-Year Hospital Volume on Surgical Margin and Biochemical-Failure-Free Survival in Patients Undergoing Robotic versus Nonrobotic Radical Prostatectomy: A Nationwide Cohort Study from the National Taiwan Cancer Database. Cancers.

[5] Salinas C.A., Tsodikov A., Ishak-Howard M., Cooney K.A. (2014). Prostate cancer in young men: An important clinical entity. Nat. Rev. Urol..

[6] Stangelberger A., Waldert M., Djavan B. (2008). Prostate cancer in elderly men. Rev. Urol..

[7] Artibani W., Fracalanza S., Cavalleri S., Iafrate M., Aragona M., Novara G., Gardiman M., Ficarra V. (2008). Learning curve and preliminary experience with da Vinci-assisted laparoscopic radical prostatectomy. Urol. Int..

[8] Hakenberg O.W. (2018). A brief overview of the development of robot-assisted radical prostatectomy. Arab. J. Urol..

[9] Walsh P.C. (2003). Prospective comparison of radical retropubic prostatectomy and robot-assisted anatomic prostatectomy: The Vattikuti Urology Institute experience. J. Urol..

[10] Menon M., Tewari A., Baize B., Guillonneau B., Vallancien G. (2002). Prospective comparison of radical retropubic prostatectomy and robot-assisted anatomic prostatectomy: The Vattikuti Urology Institute experience. Urology.

[11] Hu J.C., Gu X., Lipsitz S.R., Barry M.J., D’Amico A.V., Weinberg A.C., Keating N.L. (2009). Comparative effectiveness of minimally invasive vs open radical prostatectomy. JAMA.

[12] Ilic D., Evans S.M., Allan C.A., Jung J.H., Murphy D., Frydenberg M. (2017). Laparoscopic and robotic-assisted versus open radical prostatectomy for the treatment of localised prostate cancer. Cochrane Database Syst. Rev..

[13] Trinh Q.D., Sammon J., Sun M., Ravi P., Ghani K.R., Bianchi M., Jeong W., Shariat S.F., Hansen J., Schmitges J. (2012). Perioperative outcomes of robot-assisted radical prostatectomy compared with open radical prostatectomy: Results from the nationwide inpatient sample. Eur. Urol..

[14] Asimakopoulos A.D., Pereira Fraga C.T., Annino F., Pasqualetti P., Calado A.A., Mugnier C. (2011). Randomized comparison between laparoscopic and robot-assisted nerve-sparing radical prostatectomy. J. Sex. Med..

[15] Novara G., Ficarra V., Rosen R.C., Artibani W., Costello A., Eastham J.A., Graefen M., Guazzoni G., Shariat S.F., Stolzenburg J.U. (2012). Systematic review and meta-analysis of perioperative outcomes and complications after robot-assisted radical prostatectomy. Eur. Urol..

[16] Tewari A., Sooriakumaran P., Bloch D.A., Seshadri-Kreaden U., Hebert A.E., Wiklund P. (2012). Positive surgical margin and perioperative complication rates of primary surgical treatments for prostate cancer: A systematic review and meta-analysis comparing retropubic, laparoscopic, and robotic prostatectomy. Eur. Urol..

[17] Lim S.K., Kim K.H., Shin T.Y., Rha K.H. (2013). Current status of robot-assisted laparoscopic radical prostatectomy: How does it compare with other surgical approaches?. Int. J. Urol..

[18] Moran P.S., O’Neill M., Teljeur C., Flattery M., Murphy L.A., Smyth G., Ryan M. (2013). Robot-assisted radical prostatectomy compared with open and laparoscopic approaches: A systematic review and meta-analysis. Int. J. Urol..

[19] Porpiglia F., Morra I., Lucci Chiarissi M., Manfredi M., Mele F., Grande S., Ragni F., Poggio M., Fiori C. (2013). Randomised controlled trial comparing laparoscopic and robot-assisted radical prostatectomy. Eur. Urol..

[20] Robertson C., Close A., Fraser C., Gurung T., Jia X., Sharma P., Vale L., Ramsay C., Pickard R. (2013). Relative effectiveness of robot-assisted and standard laparoscopic prostatectomy as alternatives to open radical prostatectomy for treatment of localised prostate cancer: A systematic review and mixed treatment comparison meta-analysis. BJU Int..

[21] Tomaszewski J.J., Matchett J.C., Davies B.J., Jackman S.V., Hrebinko R.L., Nelson J.B. (2012). Comparative hospital cost-analysis of open and robotic-assisted radical prostatectomy. Urology.

[22] Graefen M. (2010). Editorial comment on: Cost comparison of robotic, laparoscopic and open radical prostatectomy for prostate cancer. Eur. Urol..

[23] Lotan Y., Cadeddu J.A., Gettman M.T. (2004). The new economics of radical prostatectomy: Cost comparison of open, laparoscopic and robot assisted techniques. J. Urol..

[24] Bolenz C., Gupta A., Hotze T., Ho R., Cadeddu J.A., Roehrborn C.G., Lotan Y. (2010). Cost comparison of robotic, laparoscopic, and open radical prostatectomy for prostate cancer. Eur. Urol..

[25] Close A., Robertson C., Rushton S., Shirley M., Vale L., Ramsay C., Pickard R. (2013). Comparative cost-effectiveness of robot-assisted and standard laparoscopic prostatectomy as alternatives to open radical prostatectomy for treatment of men with localised prostate cancer: A health technology assessment from the perspective of the UK National Health Service. Eur. Urol..

[26] Chiang C.J., You S.L., Chen C.J., Yang Y.W., Lo W.C., Lai M.S. (2015). Quality assessment and improvement of nationwide cancer registration system in Taiwan: A review. Jpn. J. Clin. Oncol..

[27] Wen C.P., Tsai S.P., Chung W.S. (2008). A 10-year experience with universal health insurance in Taiwan: Measuring changes in health and health disparity. Ann. Int. Med..

[28] Wu S.Y., Fang S.C., Hwang O.R., Shih H.J., Shao Y.J. (2020). Influence of Baseline Cardiovascular Comorbidities on Mortality after Androgen Deprivation Therapy for Metastatic Prostate Cancer. Cancers.

[29] Shia B.C., Qin L., Lin K.C., Fang C.Y., Tsai L.L., Kao Y.W., Wu S.Y. (2020). Outcomes for Elderly Patients Aged 70 to 80 Years or Older with Locally Advanced Oral Cavity Squamous Cell Carcinoma: A Propensity Score-Matched, Nationwide, Oldest Old Patient-Based Cohort Study. Cancers.

[30] Qin L., Chen T.-M., Kao Y.-W., Lin K.-C., Yuan K.S.-P., Wu A.T.H., Shia B.-C., Wu S.-Y. (2018). Predicting 90-Day Mortality in Locoregionally Advanced Head and Neck Squamous Cell Carcinoma after Curative Surgery. Cancers.

[31] Lin W.C., Chang C.L., Hsu H.L., Yuan K.S., Wu A.T.H., Wu S.Y. (2019). Three-Dimensional Conformal Radiotherapy-Based or Intensity-Modulated Radiotherapy-Based Concurrent Chemoradiotherapy in Patients with Thoracic Esophageal Squamous Cell Carcinoma. Cancers.

[32] Chang C.L., Yuan K.S., Wu A.T.H., Wu S.Y. (2019). Adjuvant Therapy for High-Risk Stage II or III Colon Adenocarcinoma: A Propensity Score-Matched, Nationwide, Population-Based Cohort Study. Cancers.

[33] Chang C.L., Yuan K.S., Wu A.T.H., Wu S.Y. (2019). Toxicity Profiles of Fractionated Radiotherapy, Contemporary Stereotactic Radiosurgery, and Transsphenoidal Surgery in Nonfunctioning Pituitary Macroadenomas. Cancers.

[34] Rivers P.A., Glover S.H. (2008). Health care competition, strategic mission, and patient satisfaction: Research model and propositions. J. Health Organ. Manag..

[35] Hung C.F., Yang C.K., Ou Y.C. (2016). Urologic cancer in Taiwan. Jpn. J. Clin. Oncol..

[36] Lepor H. (2005). A review of surgical techniques for radical prostatectomy. Rev. Urol..

[37] National Comprehensive Cancer Network NCCN Clinical Practice Guidelines in Oncology. http://www.nccn.org/professionals/physician_gls/f_guidelines.asp.

[38] Klein E.A., Grass J.A., Calabrese D.A., Kay R.A., Sargeant W., O’Hara J.F. (1996). Maintaining quality of care and patient satisfaction with radical prostatectomy in the era of cost containment. Urology.

[39] Sooriakumaran P., Srivastava A., Shariat S.F., Stricker P.D., Ahlering T., Eden C.G., Wiklund P.N., Sanchez-Salas R., Mottrie A., Lee D. (2014). A multinational, multi-institutional study comparing positive surgical margin rates among 22393 open, laparoscopic, and robot-assisted radical prostatectomy patients. Eur. Urol..

[40] Webster T.M., Herrell S.D., Chang S.S., Cookson M.S., Baumgartner R.G., Anderson L.W., Smith J.A. (2005). Robotic assisted laparoscopic radical prostatectomy versus retropubic radical prostatectomy: A prospective assessment of postoperative pain. J. Urol..

[41] Miller J., Smith A., Kouba E., Wallen E., Pruthi R.S. (2007). Prospective evaluation of short-term impact and recovery of health related quality of life in men undergoing robotic assisted laparoscopic radical prostatectomy versus open radical prostatectomy. J. Urol..

[42] Sharma N.L., Shah N.C., Neal D.E. (2009). Robotic-assisted laparoscopic prostatectomy. Br. J. Cancer.

[43] Liu C.L., Li C.C., Yang C.R., Yang C.K., Wang S.S., Chiu K.Y., Su C.K., Ho H.C., Cheng C.L., Ou Y.C. (2011). Trends in treatment for localized prostate cancer after emergence of robotic-assisted laparoscopic radical prostatectomy in Taiwan. J. Chin. Med. Assoc..

[44] Wu T.Y., Majeed A., Kuo K.N. (2010). An overview of the healthcare system in Taiwan. Lond. J. Prim. Care.

[45] Friethriksson J.O., Holmberg E., Adolfsson J., Lambe M., Bill-Axelson A., Carlsson S., Hugosson J., Stattin P. (2014). Rehospitalization after radical prostatectomy in a nationwide, population based study. J. Urol..

[46] Wallerstedt Lantz A., Stranne J., Tyritzis S.I., Bock D., Wallin D., Nilsson H., Carlsson S., Thorsteinsdottir T., Gustafsson O., Hugosson J. (2019). 90-Day readmission after radical prostatectomy-a prospective comparison between robot-assisted and open surgery. Scand. J. Urol..

[47] Cheng Y.W., Sung F.C., Yang Y., Lo Y.H., Chung Y.T., Li K.C. (2009). Medical waste production at hospitals and associated factors. Waste Manag..

[48] Cheng Y.W., Li K.C., Sung F.C. (2010). Medical waste generation in selected clinical facilities in Taiwan. Waste Manag..

