# Supplementary material for: Latest Comprehensive Medical Resource Consumption in Robot-Assisted versus Laparoscopic and Traditional Open Radical Prostatectomy: A Nationwide Population-Based Cohort Study
Source: Cancers (Basel). 2021 Mar 29;13(7):1564. doi: 10.3390/cancers13071564 (PMC8037789; doi:10.3390/cancers13071564)
Supplement: Supplementary file 1 [file cancers-13-01564-s001.pdf]

Supplemental Figure 1 Bar graph of generalized linear mixed model of medical reimbursement for urinary diseases or surgical complications stratified by open, laparoscopic, and robotic RP

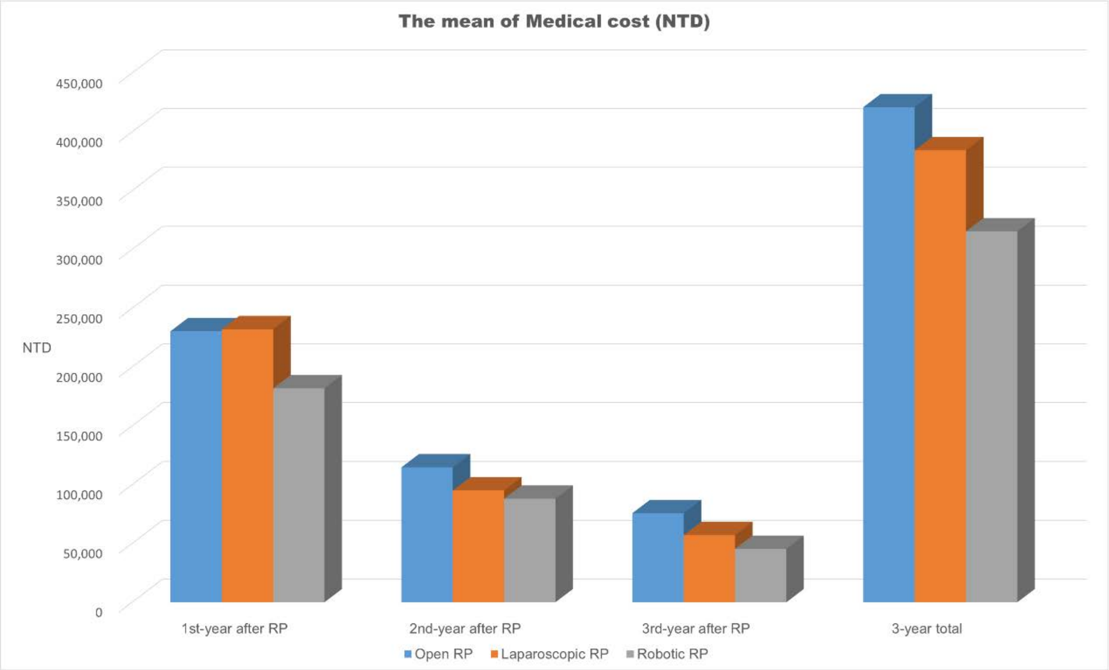

RP, radical prostatectomy; NTD, New Taiwan Dollars
